# Supplementary material for: Perilipin 3 promotes the formation of membrane domains enriched in diacylglycerol and lipid droplet biogenesis proteins
Source: Front Cell Dev Biol. 2023 Jul 3;11:1116491. doi: 10.3389/fcell.2023.1116491 (PMC10350540; doi:10.3389/fcell.2023.1116491)
Supplement: Supplementary file 1 [file DataSheet1.pdf]

Supplementary Tables

Table S1. *Saccharomyces cerevisiae* strains used in this study

Name	*Relevant Genotype*	Source
RSY 5669	*MATα his3Δ1 leu2Δ0 ura3Δ0 are1Δ::KanMX are2Δ::KanMX dga1Δ::KanMX lro1Δ::KanMX lys2Δ*	Lab Collection
RSY 6245	*MATα his3Δ1 leu2Δ0 lys2Δ0 ura3Δ0 ERG6-mCherry::HIS3*	Will Prinz
RSY 6249	*MATα his3Δ1 leu2Δ0 lys2Δ0 ura3Δ0 are1Δ::KanMX are2Δ::KanMX dga1Δ::lox-HIS-lox trp1::URA3 GAL-LRO1::TRP1 ERG6-mCherry::HIS3*	Will Prinz
RSY 6372	*MATα his3Δ1 leu2Δ0 lys2Δ0 ura3Δ0 are1Δ::KanMX, are2Δ::KanMX lro1Δ::TRP1 dga1Δ::loxP FLD1-GFP::HIS3*	Lab Collection
RSY 7185	*MATa his3Δ1 leu2Δ0 ura3Δ0 are1Δ::KanMX are2Δ::KanMX dga1Δ::KanMX lro1Δ::KanMX lys2Δ fld1Δ::NAT*	This Study
RSY 7188	*MATa his3Δ1 leu2Δ0 ura3Δ0 are1Δ::KanMX are2Δ::KanMX dga1Δ::KanMX lro1Δ::KanMX lys2Δ FLD1-mScarlet::HIS3*	This Study
RSY 7189	*MATa his3Δ1 leu2Δ0 ura3Δ0 are1Δ::KanMX are2Δ::KanMX dga1Δ::KanMX lro1Δ::KanMX lys2Δ TGL1-GFP::HIS3*	This Study
RSY 7190	*MATa his3Δ1 leu2Δ0 ura3Δ0 are1Δ::KanMX are2Δ::KanMX dga1Δ::KanMX lro1Δ::KanMX lys2Δ PEX30-mScarlet::HIS3*	This Study
RSY 7216	*MATa his3Δ1 leu2Δ0 ura3Δ0 are1Δ::KanMX are2Δ::KanMX dga1Δ::KanMX lro1Δ::KanMX lys2Δ ldb16Δ::NAT*	Lab Collection
RSY 7227	*MATa his3Δ1 leu2Δ0 ura3Δ0 are1Δ::KanMX are2Δ::KanMX dga1Δ::KanMX lro1Δ::KanMX lys2Δ ldo16/45Δ::NAT*	Lab Collection
RSY 7259	*MATa his3Δ1 leu2Δ0 ura3Δ0 are1Δ::KanMX are2Δ::KanMX dga1Δ::KanMX lro1Δ::KanMX lys2Δ LDO-GFP::HIS3*	Lab Collection
RSY 7262	*MATa his3Δ1 leu2Δ0 ura3Δ0 are1Δ::KanMX are2Δ::KanMX dga1Δ::KanMX lro1Δ::KanMX lys2Δ LDB16-GFP::HIS3*	Lab Collection
RSY 7336	*MATa his3Δ1 leu2Δ0 ura3Δ0 are1Δ::KanMX are2Δ::KanMX dga1Δ::KanMX lro1Δ::KanMX lys2Δ nem1Δ::HIS3*	This Study

RSY 7358	*MATa his3Δ1 leu2Δ0 ura3Δ0 are1Δ::KanMX are2Δ::KanMX dga1Δ::KanMX lro1Δ::KanMX lys2Δ NEM1-mScarlet::HIS3*	This Study
RSY 8042	*MATa his3Δ1 leu2Δ0 ura3Δ0 are1Δ::KanMX are2Δ::KanMX dga1Δ::KanMX lro1Δ::KanMX lys2Δ GFP-IST2::URA3*	This Study
RSY 7360	*MATa his3Δ1 leu2Δ0 ura3Δ0 are1Δ::KanMX are2Δ::KanMX dga1Δ::KanMX lro1Δ::KanMX lys2Δ SPO7-mScarlet::HIS3*	This Study

Table S2. Plasmids used in this study

Plasmids	Source
pCM189-tetO$_7$-PLIN3-GFP/URA3	Khaddaj et al., 2022
pCM189-tetO$_7$-WBP1-GFP-PLIN3/ URA3	Khaddaj et al., 2022
pCM189-tetO$_7$-SEC61-GFP-PLIN3/ URA3	Khaddaj et al., 2022
pCM189-tetO$_7$-WBP1-GFP-PLIN3/ HIS3	This Study
pCM189-tetO$_7$-SEC61-GFP-PLIN3/ HIS3	This Study
pCM189-tetO$_7$-WBP1-GFP/ URA3	Khaddaj et al., 2022
pCM189-tetO$_7$-SEC61-GFP/ URA3	Khaddaj et al., 2022
pCM189-tetO$_7$-WBP1-mScarlet-PLIN3/ URA3	Khaddaj et al., 2022
pCM189-tetO$_7$-SEC61-mScarlet-PLIN3/ URA3	Khaddaj et al., 2022
pCM189-tetO$_7$-WBP1-mScarlet-PLIN3/ LEU2	This Study
pCM189-tetO$_7$-SEC61-mScarlet-PLIN3/ LEU2	This Study
pCM189-tetO$_7$-WBP1-mScarlet/ URA3	Khaddaj et al., 2022
pCM189-tetO$_7$-SEC61-mScarlet/ URA3	Khaddaj et al., 2022
pRS415-ADH-mCherry-HDEL/ LEU2	Lab Collection
pCM189-tetO$_7$-WBP1-GFP-PLIN3^{V158D}/ URA3	This Study
pCM189-tetO$_7$-SEC61-GFP-PLIN3^{V158D}/ URA3	This Study
pCM189-tetO$_7$-WBP1-mScarlet-PLIN1/ URA3	This Study
pCM189-tetO$_7$-SEC61-mScarlet-PLIN1/ URA3	This Study
pCM189-tetO$_7$-WBP1-mScarlet-PAT/ URA3	This Study
pCM189-tetO$_7$-WBP1-mScarlet-11-mer/ URA3	This Study
pCM189-tetO$_7$-WBP1-mScarlet-4-helix bundle/ URA3	This Study
pCM189-tetO$_7$-WBP1-mScarlet-PAT+11-mer/ URA3	This Study
pCM189-tetO$_7$-WBP1-mScarlet-11-mer+4-helix bundle/ URA3	This Study
pCM189-tetO$_7$-WBP1-mScarlet-PAT+4-helix bundle/ URA3	This Study
pCM189-tetO$_7$-SEC61-mScarlet-PAT/ URA3	This Study
pCM189-tetO$_7$-SEC61-mScarlet-11-mer/ URA3	This Study
pCM189-tetO$_7$-SEC61-mScarlet-4-helix bundle/ URA3	This Study
pCM189-tetO$_7$-SEC61-mScarlet-PAT+11-mer/ URA3	This Study
pCM189-tetO$_7$-SEC61-mScarlet-11-mer+4-helix bundle/ URA3	This Study

pCM189-tetO$_7$-SEC61-mScarlet-PAT+4-helix bundle/ URA3	This Study
pYEplac181-ADH-ER-DAG-Sensor/ LEU2	Will Prinz
pYEplac181-ADH-Mutated-ER-DAG-Sensor/ LEU2	Will Prinz
pRS416-ADH-TGL4-GFP/ URA3	Lab Collection
pRS415-GAL-AYR1-GFP/ LEU2	Lab Collection
pGREG503-PAH1-7A/ HIS3	Lab Collection

Supplementary References

Khaddaj, R. et al. (2022). The surface of lipid droplets constitutes a barrier for endoplasmic reticulum-resident integral membrane proteins. J Cell Sci, 135, jcs256206. 10.1242/jcs.256206

Supplementary Figure Legends

Figure S1. Comparison of the subcellular localization of free GFP to that of PLIN3-GFP and the ER-DAG sensor in cells lacking or containing LDs.

Wild-type and cells lacking LDs (*4*Δ) expressing either free GFP, GFP fused to PLIN3, or the ER-DAG sensor were cultivated, and the subcellular distribution of the fluorophore was analyzed by confocal microscopy. Scale bar, 5 µm.

Figure S2. Fusion of PLIN1 to Wbp1 or Sec61 membrane anchors also induces formation of ER domains that colocalize with BODIPY or with the ER-DAG sensor

A) A mutated ER-DAG sensor does not concentrate at ER crescents. A point mutant version of the GFP-tagged ER-DAG sensor (P155G) was expressed in *4*Δ cells coexpressing either soluble mScarlet-PLIN3 or its membrane-anchored versions. Cells were cultivated and the localization of the DAG-sensor was analyzed by confocal microscopy. ER domains formed by expression of the membrane-anchored PLIN3 are indicated by white arrowheads, staining of the perinuclear ER by the mutated ER-DAG sensor is indicated by blue arrows. Scale bar, 5 µm.

B) Doxycycline-dependent induction of Wbp1-GFP-PLIN3 and Sec61-GFP-PLIN3. Cells were cultivated overnight in the presence of doxycycline. The repressor was washed off at time 0 and samples collected at the indicated time points were analyzed by Western blotting to monitor time-dependent changes in expression levels of the fusion proteins.

C) Quadruple mutant cells (*4*Δ*; are1*Δ *are2*Δ *dga1*Δ *lro1*Δ) expressing Wbp1-GFP-PLIN1 or Sec61-GFP-PLIN1 were stained with BODIPY and the distribution of the fluorescent-tagged proteins was analyzed by confocal microscopy. The ER domains formed by expression of the membrane-anchored PLIN1 are indicated by white arrowheads, co-staining by BODIPY is indicated by blue arrows. Scale bar, 5 µm.

D) Quadruple mutant cells coexpressing Wbp1-mScarlet-PLIN1 or Sec61-mScarlet-PLIN1 with the GFP-tagged ER-DAG sensor were cultivated. The distribution of the marker proteins was analyzed by confocal microscopy. ER domains formed by membrane-anchored PLIN1 are indicated by white arrowheads, that of the ER-DAG sensor by blue arrows. Scale bar, 5 µm.

E) DAG levels decline upon treatment of cells with terbinafine and cerulenin. Cells were cultivated overnight, diluted and treated (T) or not treated (U) with the sterol synthesis inhibitor terbinafine (30 µg/ml) or the fatty acid synthase inhibitor cerulenin (10 µg/ml) for 2 h. Lipids were extracted, spiked

with an internal standard (C17:0 DAG) and quantified by mass spectrometry. Levels of untreated samples were normalized to 100%.

Figure S3. Seipin colocalizes with ER domains formed by membrane-anchored PLIN3 two-domain fusions

A, B) Quadruple mutant cells expressing GFP-tagged seipin (Fld1) and the indicated Wbp1- (panel A) or Sec61- (panel B) based PLIN3 double domain fusions were imaged by confocal microscopy. Membrane domains formed by the membrane-anchored PLIN double domain fusions are indicated by white arrowheads, foci formed by seipin are indicated by orange arrow heads. Scale bar, 5 µm.

Figure S4. Membrane-anchored two PLIN3 protein domain fusions colocalize with LDs in wild-type cells

A, B) Wild-type cells expressing the indicated membrane-anchored PLIN3 single or double protein domain fusions were incubated with BODIPY to stain LDs and imaged by confocal microscopy. Localization of the membrane-anchored PLIN3 fusions is indicated by white arrowheads, BODIPY stained LDs are indicated by blue arrows. Scale bar, 5 µm.

C, D) Quantification of colocalization between the membrane-anchored PLIN3 domain reporter and BODIPY stained LDs. Colocalization was scored manually by pixel analysis, data represent mean ± S.D. of n=50 cells.

Figure S5. Specificity of short chain lipid binding by PLIN3 and determination of optimal levels of DAG in liposomes

A) PLIN3 does not bind short chain phosphatidylinositol (PI). Binding of PLIN3 to C8:0 PI was assessed by MST in three independent measurements. No dissociation constant (K_D) could be determined (n.d.).

B) Binding of PLIN3 to DOPC/DOPE-liposomes (80/20 mol%) containing increasing concentration of C16:0 DAG. Dissociation constants are indicated above the corresponding bars. Values represent mean ± S.D. of three independent determinations. Statistical analysis was performed using an unpaired *t*-test (* $p<0.05$; ** $p<0.01$).

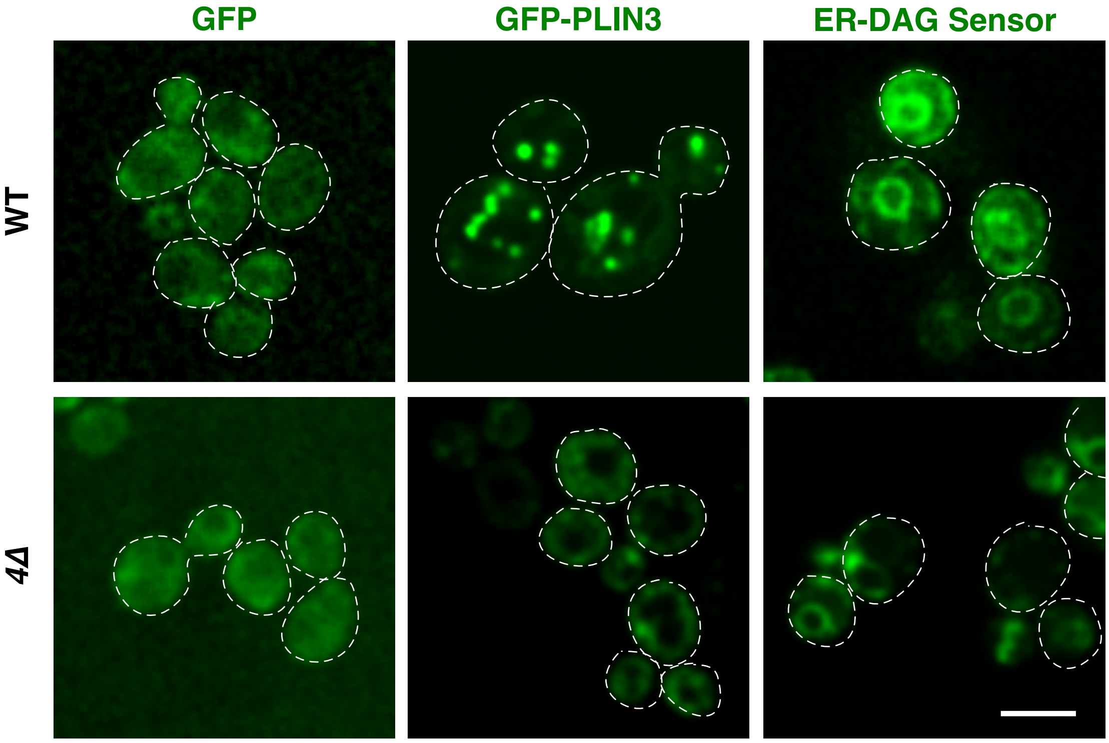

Figure S1

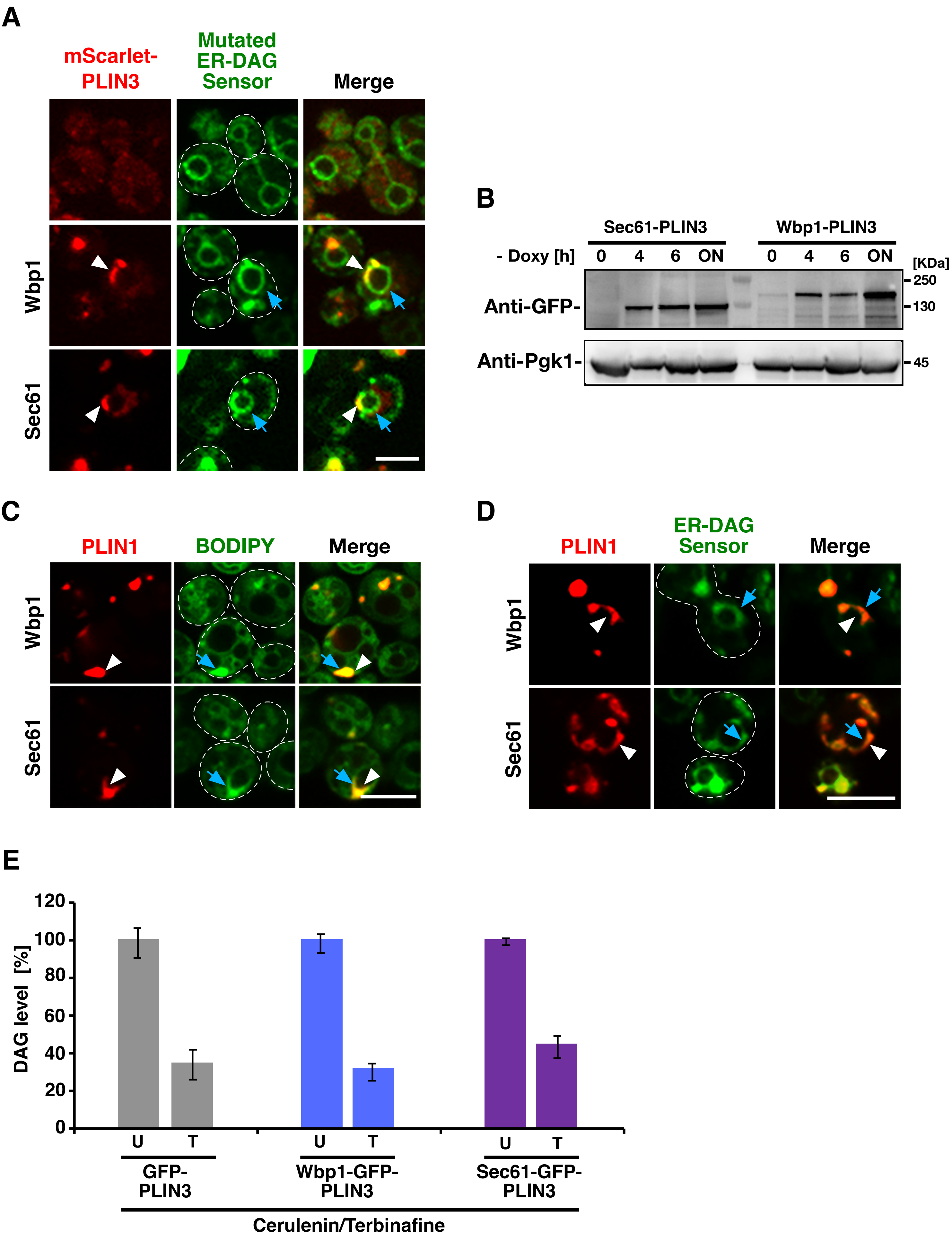
A
mScarlet-PLIN3
Mutated ER-DAG Sensor
Merge
Wbp1
Sec61
B
Sec61-PLIN3
Wbp1-PLIN3
- Doxy [h]
0
4
6
ON
0
4
6
ON
[KDa]
250
130
45
Anti-GFP-
Anti-Pgk1-
C
PLIN1
BODIPY
Merge
Wbp1
Sec61
D
PLIN1
ER-DAG Sensor
Merge
Wbp1
Sec61
E
DAG level [%]
120
100
80
60
40
20
0
U
T
U
T
U
T
GFP-PLIN3
Wbp1-GFP-PLIN3
Sec61-GFP-PLIN3
Cerulenin/Terbinafine

Figure S2

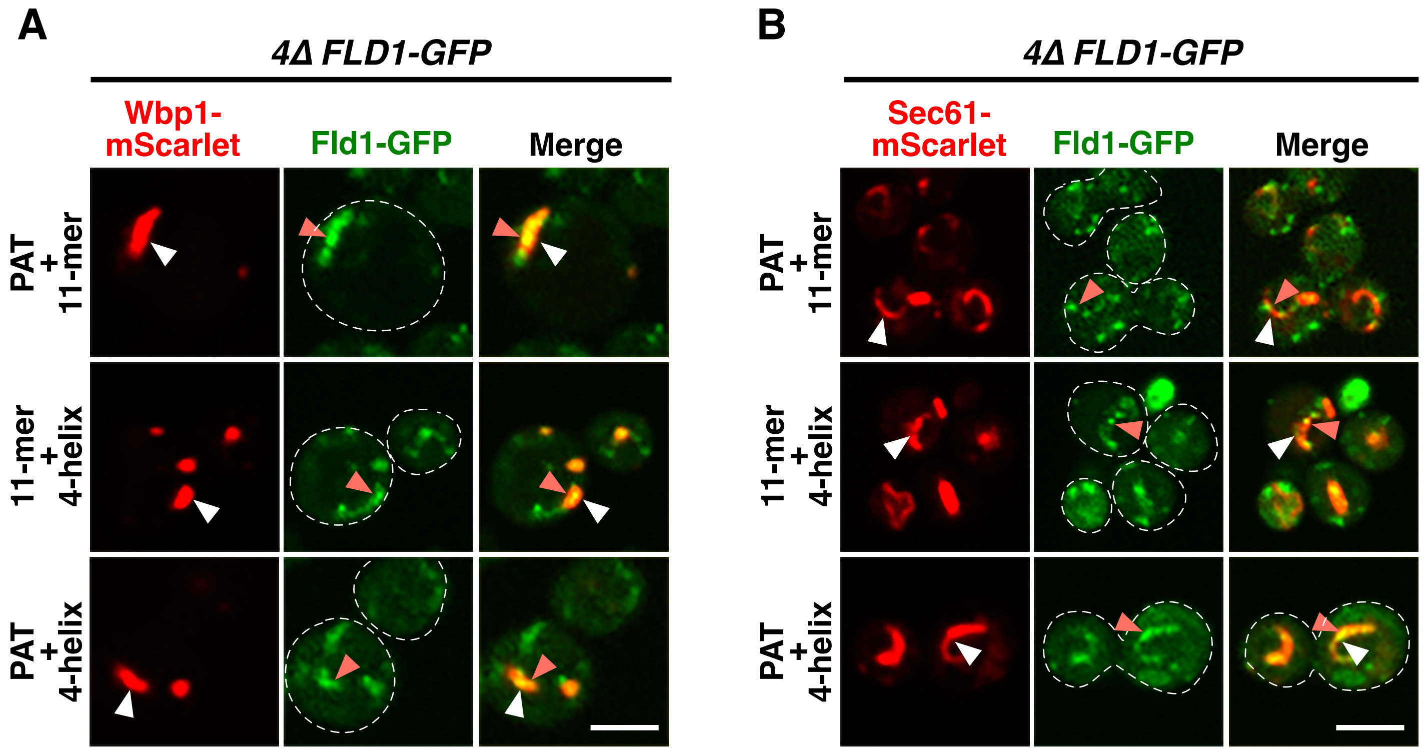
A
4Δ FLD1-GFP
Wbp1-mScarlet
Fld1-GFP
Merge
PAT + 11-mer
11-mer + 4-helix
PAT + 4-helix
B
4Δ FLD1-GFP
Sec61-mScarlet
Fld1-GFP
Merge
PAT + 11-mer
11-mer + 4-helix
PAT + 4-helix

Figure S3

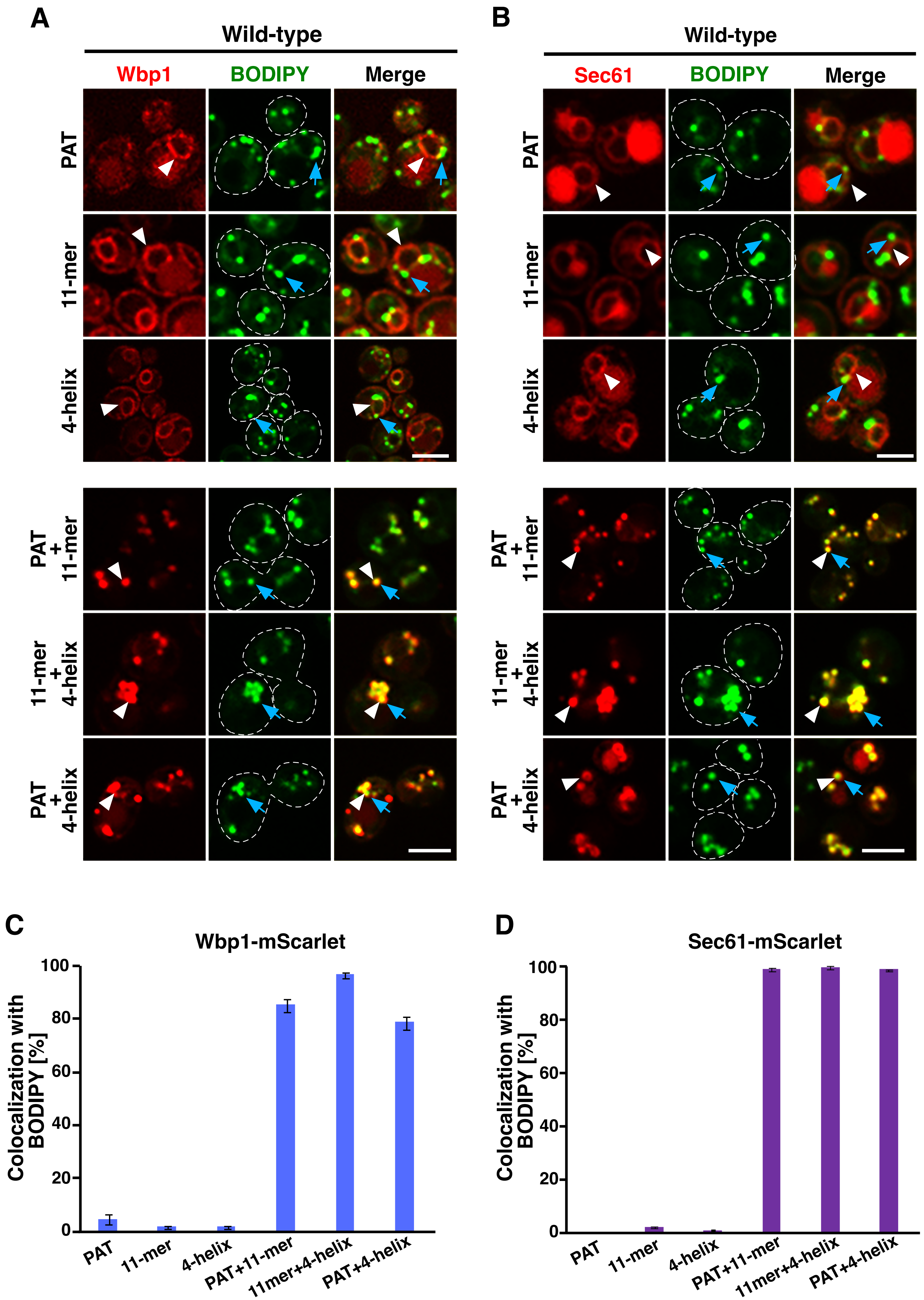
A
Wild-type
Wbp1
BODIPY
Merge
PAT
11-mer
4-helix
PAT + 11-mer
11-mer + 4-helix
PAT + 4-helix
B
Wild-type
Sec61
BODIPY
Merge
C
Wbp1-mScarlet
Colocalization with BODIPY [%]
100
80
60
40
20
0
PAT
11-mer
4-helix
PAT+11-mer
11mer+4-helix
PAT+4-helix
D
Sec61-mScarlet

Figure S4

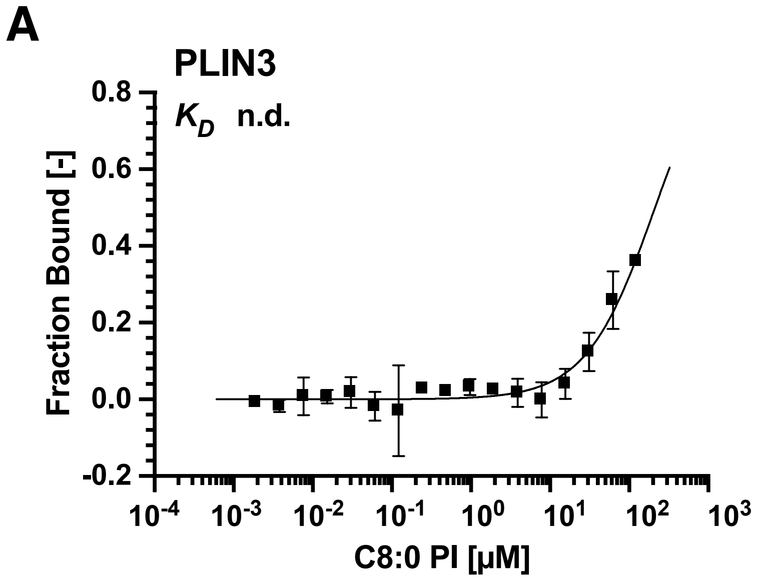
A
PLIN3
K_D n.d.
Fraction Bound [-]
0.8
0.6
0.4
0.2
0.0
-0.2
10^{-4} 10^{-3} 10^{-2} 10^{-1} 10^{0} 10^{1} 10^{2} 10^{3}
C8:0 PI [µM]

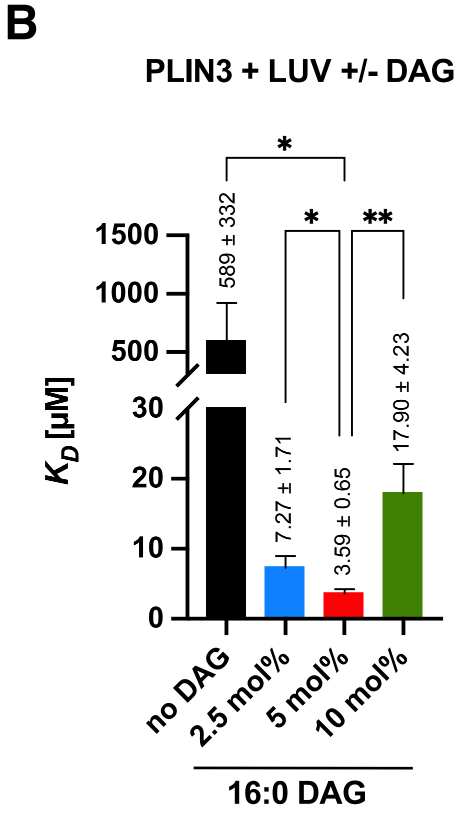
B
PLIN3 + LUV +/- DAG
*
*
**
589 ± 332
7.27 ± 1.71
3.59 ± 0.65
17.90 ± 4.23
K_D [µM]
1500
1000
500
30
20
10
0
no DAG
2.5 mol%
5 mol%
10 mol%
16:0 DAG

Figure S5
